# Postpartum perineal muscle sonogram in Madura beef cow

**DOI:** 10.14202/vetworld.2021.380-392

**Published:** 2021-02-10

**Authors:** Sari Yanti Hayanti, Amrozi Amrozi, Aryogi Aryogi, Mokhamad Fakhrul Ulum

**Affiliations:** 1 Study Program of Reproductive Biology, Postgraduate School, Bogor Agricultural University, Bogor, West Java of Indonesia; 2 Department of Resource Research, Assessment Institute for Agricultural Technology of Jambi, Indonesian Agency for Agricultural Research and Development, Ministry of Agriculture, Jambi, Jambi of Indonesia; 3 Division of Veterinary Reproduction, Obstetrics and Gynecology, Department of Veterinary Clinic, Reproduction, and Pathology, Faculty of Veterinary Medicine, Bogor Agricultural University, Bogor, West Java of Indonesia; 4 Department of Livestock Breeding, Beef Cattle Research Institute, Pasuruan East Java, Indonesian Center for Animal Research and Development, Indonesian Agency for Agricultural Research and Development, Ministry of Agriculture, Grati, Pasuruan, East Java of Indonesia

**Keywords:** coccygeus, levator ani, Madura beef cow, postpartum, ultrasonography

## Abstract

**Background and Aim:**

Ultrasonography (USG) is useful for non-invasively identifying changes that occur in soft tissue architecture. The objective of this research was to demonstrate postpartum (PP) uterine involution through the changes of perineal muscle intensity and thickness in Madura beef cow by ultrasonography.

**Materials and Methods:**

Madura’s breed cows used in the research consist of eight non-pregnant (NP) cows and three PP cow. The transrectal and transperineal USG imaging of NP cows was performed on days 1, 33, and 65. USG imaging of PP cows was performed every day starting from day 1 (24 h after parturition) until day 21 PP. Transrectal USG of the reproductive tract was performed for the cervix, corpus uteri, and cornua uteri. USG was performed transcutaneously over the perineal area for coccygeus and levator ani muscles at the longitudinal and transverse angles. Reproductive tract diameter and perineal muscle intensity and thickness were measured with ultrasound imaging.

**Results:**

The analysis of the sonogram of PP cows showed that the diameter of the cervix, corpus uteri, and cornua uteri decreased within 21 days PP. The transverse view of the coccygeus muscle of PP cows showed decreased muscle intensity and thickness. On the other hand, the longitudinal view showed increased coccygeus muscle intensity and thickness. The transverse view of the coccygeus muscle of NP cows showed increased muscle intensity, while muscle thickness was reduced. Sonogram analysis of the levator ani muscle of PP cows showed decreased muscle intensity with increasing muscle thickness. However, imaging of the levator ani muscle of NP cows showed a decrease in both intensity and muscle thickness. There was a significant difference in the mean value intensity of the scanning view analysis results of the levator ani muscle of the PP cow (523.6 AU increased to 672.1 AU) and the NP cow (515.9 AU decreased to 465.4 AU). Furthermore, there was a significant difference (p<0.05) in the mean value thickness of both scanning view analyses of the coccygeus and levator ani muscles of PP cows (5.8 mm increased to 6.5 mm and 3.8 mm increased to 4.8 mm, respectively) and NP cows (8.8 mm increased to 9.1 mm and 5.9 mm decreased to 4.9 mm, respectively).

**Conclusion:**

We found that the perineal muscles, namely, the levator ani muscle and coccygeus muscle, change in size, and intensity during uterine involution as demonstrated on Madura beef cow.

## Introduction

Ultrasonography (USG) is an imaging technology based on ultra-high frequency sound waves that is commonly used in the medical field for both human and animal subjects. USG technology has been used in reproduction to observe ovarian activity [1], early pregnancy [2], reproductive disorders [3], and postpartum (PP) changes in the reproductive tract [4]. USG imaging has been used to observe the uterine involution process of Friesian Holstein (FH) cows [5], Ongole crossbreed cows [6], and water buffaloes [7]. Complete and fast uterine involution will result in early resumption of the estrous cycle and better PP reproductive performance [8].

Hind region (perineal) muscles in humans and animals, such as cows, undergo relaxation during the birth process due to relaxin hormone [9,10]. It has been reported that the recovery of the perineal muscle must be monitored intensively in humans during PP to the purpureum period [11]. This also needs to be done in cows to determine the restoration process of the involuted perineal muscles of the reproductive organs of the cow. Recovery of the perineal muscle PP can clearly be imaged by USG to observe the muscle condition [12]. The same imagery used in livestock has been performed in FH cows to differentiate muscle intensity and thickness between cows in cycle, gestating cows, and cows in purpureum period [13]. Information on these changes is also needed to determine whether there is a change in the perineal muscles during uterine involution in beef cows. One of the local beef cows in Indonesia with good reproductive performance is the Madura cattle [14].

Changes in the reproductive organs during the involution process will influence the intensity and thickness of the perineal muscle. Uterine weight decreases from almost 9 kg PP to 1 kg on day 30 PP in FH cow [15]. The rate of change of perineal muscle intensity and thickness through dynamic USG imaging can be used to observe uterine involution. The changing muscle conditions can predict the efficiency of energy consumption in the body during the PP period allowing the optimization of nutritional requirements of PP cows, which can influence the reproductive performance quality of the cow during the next period [16].

This research aimed to determine the changes in intensity and thickness of the perineal muscles (coccygeus muscle and levator ani) in PP Madura beef cow using brightness-mode (B-mode) ultrasonography.

## Materials and Methods

### Ethical approval

This research has received approval from Test Animal Welfare Committee (Komisi Kesejahteraan Hewan Coba-KKHB), Agricultural Research and Development Agency under Number: Balitbangtan/Lolitsapi/Rm/16/2019.

### Study period and location

This research was conducted from November 2, 2019, to February 1, 2020, at Beef Cattle Research Institute, Grati, Pasuruan, East Java of Indonesia.

### Animals

The Madura cattle used consist of eight non-pregnant (NP) cows and three PP cows. Cows were observed to have passed 2-3 breastfeeding time periods with the body condition score (BCS) group on a scale of 2.5-3.5 (scale 5). Cows were reared with the same feed formulation and forage, which was 10% of their body weight, and water were given *ad libitum*.

### Transrectal ultrasonography

Tools used were an SIUI CTS-900V ultrasound console with linear probe in 5.0 MHz frequency, Asus 200M× notebook, livestock handling equipment, electric shaving tool, plastic glove, and USG gel. Transrectal and transperineal USG of NP cows were performed on days 1, 33, and 65, while in PP cows, USG was performed daily starting from day 1 (24 h PP) up to day 21 PP. Transrectal USG of Madura beef cow began after evacuating feces from the rectum and exploring the position of the reproductive organs. USG was performed on the cervix, uterine corpus, and uterine cornua with the probe in the longitudinal position. The imaging depth for NP and PP was 63 mm and 87 mm, respectively, while the image width for both was 65 mm. The obtained sonogram file was in JPG format. The sonogram file was transferred from the ultrasonographic monitor to a notebook, without undergoing any changes in resolution.

### Transcutaneous ultrasonography

**Figure-1:**
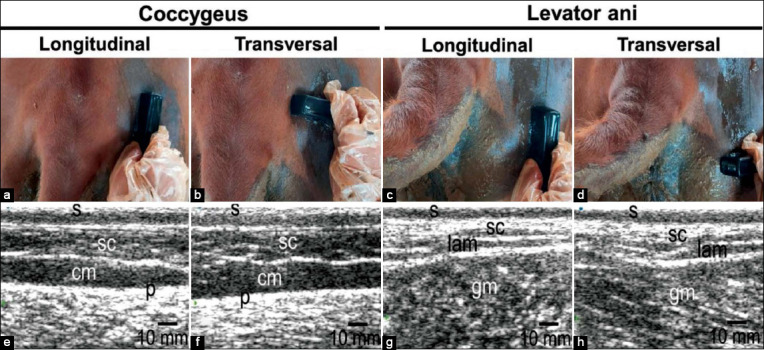
Perineal muscle and reproductive tract imaging in Madura beef cow. (a) Ultrasonography (USG) imaging of the coccygeus muscle in the longitudinal view. (b) USG imaging of the coccygeus muscle in the transverse view. (c) USG imaging of the levator ani muscle in the longitudinal view. (d) USG imaging of the levator ani muscle in the transverse. (e) Sonogram of the coccygeus muscle in the longitudinal view. (f) Sonogram of the coccygeus muscle from the transverse view. (g) Sonogram of the levator ani in the longitudinal view, and (h) sonogram of the levator ani muscle in the transverse view. s=Skin, sc=Subcutaneous, cm=Coccygeus muscle, lam=Levator ani muscle, p=Peritoneum, and gm=Gluteus muscle.

Transcutaneous USG of the perineal area was performed on the right side of coccygeus and levator ani muscles in the longitudinal and transverse plane. Transperineal USG was performed after clipping and shaving hairs around the coccygeus and levator ani muscles. The shaved area was 15 cm×10 cm wide with a remaining hair length of ±0.3 mm. Gel was applied to the shaved area, followed by USG imaging. Image format was 32 mm in depth and 65 mm in width with the sonogram file obtained in JPG (Figure-1). The sonogram file was transferred from the ultrasonographic monitor to a notebook, without undergoing any changes in resolution.

### Measurement of the diameter of the reproductive organs

The reproductive organs were measured by inputting the sonograms on ImageJ (NIH, USA) software. Organ diameter is measured by uniformly adjusting the set scale to a sonogram depth of 63 mm and a unit of length of mm without changing the sonogram resolution. The measurement of the organ diameter is done by placing a straight line from one end of the outer boundary point of the reproductive organ wall to the other. Measurement of the diameter of one organ was carried out at three different places, namely at 1/4, 1/2, and 3/4 parts, then the three results were averaged.

### Measurement of muscle intensity and thickness

The muscle intensity and thickness of the perineal muscles were measured by inputting the sonogram on ImageJ (NIH, USA) software. Measurement of the intensity and diameter begins with uniformly adjusting the set scale to a sonogram depth of 32 mm and a unit of length of mm without changing the sonogram pixel (1024×768 pixel). Muscle intensity was measured by tracing a segmented line along the fascia with points at a distance of 0.03 mm encircling the muscle until the starting point is met. Muscle diameter measurement was done by drawing a straight line from the outer boundary point of one end of the muscle fascia to the other. Measurement of the diameter of one muscle was carried out at three different places, namely 1/4, 1/2, and 3/4 parts, and then the three results were averaged.

### Statistical analysis

The measured data muscle thickness and intensity using ImageJ were then tabulated using Microsoft Excel 2016. Data obtained were analyzed using SPSS version 25.0 (SPSS Inc., Chicago, IL) with one way-analysis of variance test followed by *post hoc* Duncan to determine the differences between the group, with a significance level of p<0.05.

## Results

**Figure-2:**
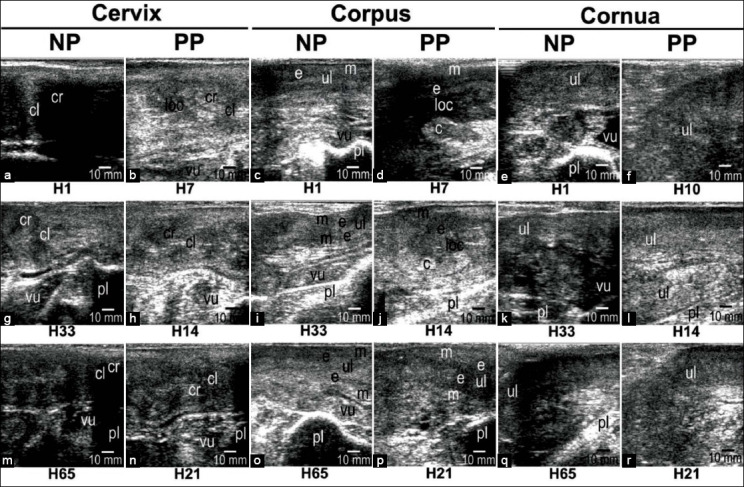
The sonogram of the reproductive tract cervix, corpus uterus, and cornua uterus in the longitudinal view of non-pregnant Madura beef cow and pregnant Madura beef cow. (a, c, e)= day 1; (b, d)=day 7; (f)=day 10; (g, i, k)=day 33; (h, j, l)=day 14; (m, o, q)=day 65; and (n, p, r)=day 21. loc=Lochia, c=Caruncle, cr=Cervix ring, cl=Cervix lumen, p=Peritoneum, e=Endometrium, m=Myometrium, ul=Uterus lumen, vu=Vesica urinaria, and pl=Pelvis.

The sonograms showing the process of involution of the reproductive tracts, consisting of the cervix, corpus uterus, and cornua uterus, in Madura beef cows are provided in Figure-2. The sonogram of the corpus uterus of the PP Madura beef cow shows caruncle shrinkage on days 7, 14, and 21, wherein caruncles were no longer visible by day 21. Other than the caruncle, the lochia appears anechoic with a wider area on day 7 compared to 14, and by day 21 lochia was no longer visible. The cornua uterus in the PP cow was not entirely visible in the sonogram on day 10, having a larger diameter compared to the day before. The cornua uterus in the longitudinal view appeared in the sonogram on day 21, wherein its diameter in the PP cow was still larger than that of the NP cow.

**Figure-3:**
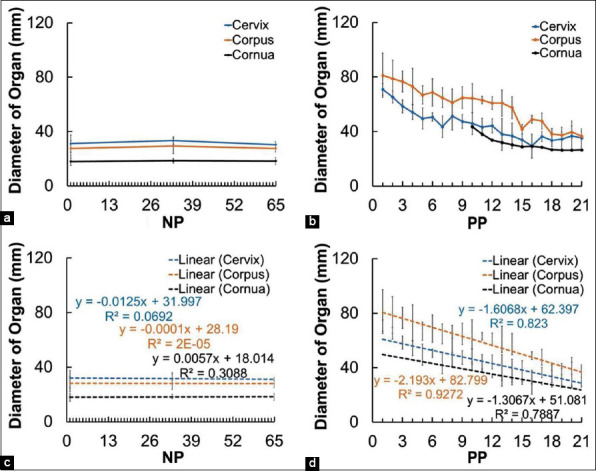
Changes in diameter and the linearity of the reproductive organ cervix, corpus uterus, and cornua uterus in non-pregnant Madura beef cow and postpartum Madura beef cow. a=NP organ diameter, b=PP organ diameter, c=Linearity of NP organ diameter, and d=linearity of PP organ.

The diameter of the cervix, corpus uterus, and cornua uterus of NP and PP Madura beef cow is in Figure-3. The uterine involution process was observed in the USG of Madura beef cow as a decrease in the diameter of the cervix, corpus uterus, and cornua uterus. The cervix, corpus uterus, and cornua uterus of the PP cow decreased in diameter from day 1 PP to day 21. Cervix diameter PP was initially 70.8 mm, and on day 21, it decreased to 35.4 mm. The corpus uterus of PP cow on day 1 PP was 81.2 mm, which decreased to 36.5 mm on day 21. The diameter of cornua uterus on day 10 PP was 43.5 mm and decreased to 26.6 mm on day 21. However, NP cow observed for 65 days did not experience significant diameter changes since day 1 of observation. The cervix of the NP cow at the beginning of observation was 31.1 mm in diameter, which decreased to 30.3 mm at the end of the observation on day 65. Of the corpus uterus diameter of the NP cow did not change from the beginning of observation until the end. The diameter of the cornua uterus of NP cow at the start of observation was 17.9 mm, which increased to 18.2 mm at the end of observation.

**Figure-4:**
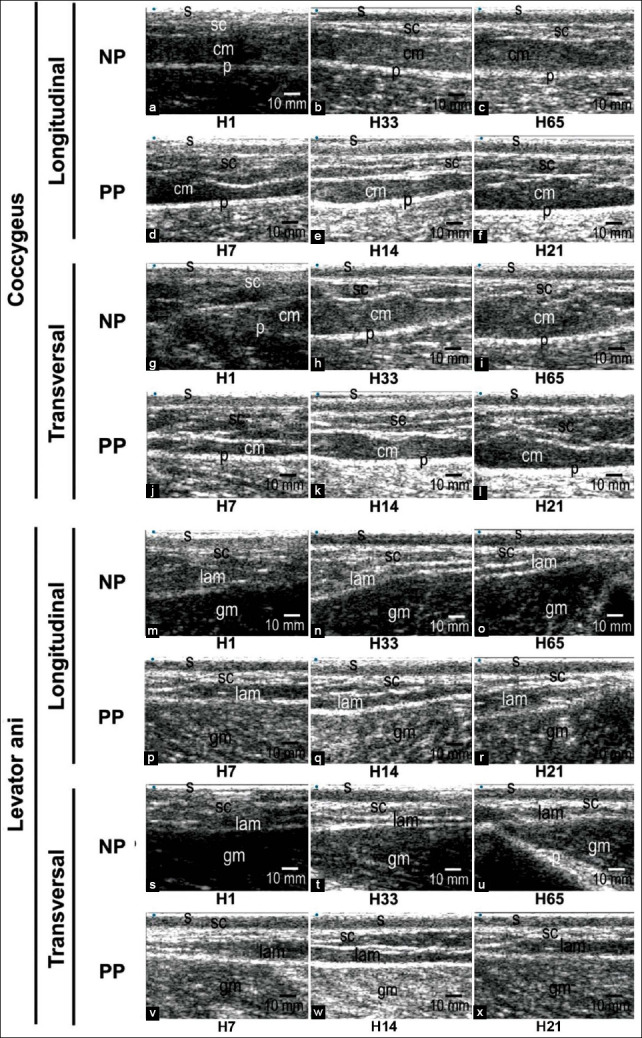
Sonogram of the coccygeus muscle and levator ani muscle of non-pregnant Madura beef cow and postpartum Madura beef cow in longitudinal and transverse views. (a, g, m, s)=day 1; (d, j, p, v)=day 7; (b, h, n, t)=day 33; (e, k, q, w)=day 14; (c, i, o, u)=day 65; and (f, l, r, x)=day 21. s=Skin, sc=Subcutaneous, cm=Coccygeus muscle, Lam=Levator ani muscle, p=Peritoneum, and gm=Gluteus muscle.

The sonogram of the coccygeus and levator ani muscles in NP and PP Madura cow is shown in Figure-4. The sonogram of the coccygeus muscle of the NP Madura beef cow in longitudinal and transverse view showed increasing echogenicity from day 1 up to 65, while the echogenicity of the levator ani muscle was visible on day 1 up to day 33 sonograms. Thereafter, the echogenicity decreased on day 65. The sonogram of the coccygeus muscle of the PP Madura beef cow on longitudinal and transverse view showed increased echogenicity but was hardly discernable. However, the sonogram of the levator ani muscle in both the longitudinal and transverse showed an increase in echogenicity on days 7-21.

**Figure-5:**
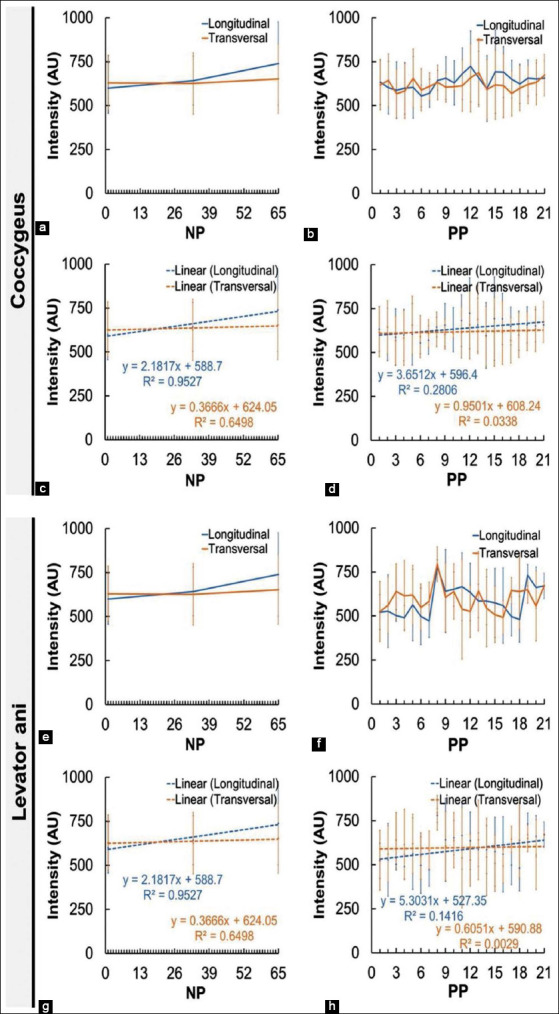
The intensity of the perineal muscles (coccygeus and levator ani muscle) in non-pregnant and postpartum Madura beef cow. a=Intensity of coccygeus muscle of NP cow, b=Intensity of coccygeus muscle of PP cow, c=Linearity of coccygeus muscle of NP cow, d=Linearity of coccygeus muscle of PP cow, e=Intensity of levator ani muscle of NP cow, f=Intensity of levator ani muscle of PP cow, g=Linearity of levator ani muscle of NP cow, and h=Linearity of levator ani muscle of PP cow.

The imaging of the coccygeus and levator ani muscle intensity in NP and PP Madura beef cow is shown in Figure-5. The intensity of the coccygeus muscle of the NP cow appeared to increase in both the longitudinal and transverse view. Intensity in the longitudinal angle on day 1 was 570.9 AU and on day 65 was 687.4 AU, whereas intensity in the transverse angle was 577.3 AU on day 1 and 653.4 AU on day 65. The intensity of the levator ani muscle in the longitudinal view appeared to increase from day 1 up to day 33, where 537.1 AU changed to 605.3 AU, which decreased to 406.9 AU by day 65. The same is found in the transverse view, where there was an increase from day 1 to day 33 from 482.9 AU to 501.3 AU, decreasing to 367.6 AU on day 65.

The intensity of the coccygeus muscle of the PP Madura beef cow in the longitudinal view decreased from 689.3 AU in day 1 to 588.5 AU in day 21. A similar case was found in the transverse view, which showed a decrease from 639.3 AU in day 1 to 578.9 AU in day 21. These results were supported by the linearity of the decrease in the longitudinal view and the transverse view. The intensity of the levator ani muscle of the PP Madura beef cow in both the longitudinal and transverse view appeared to decrease. In the longitudinal view, the intensity was 560.4 AU in day 1 and 497.3 AU in day 21. The transverse view also showed a decrease from 594.9 in day 1 to 554.5 in day 21. Although the levator ani intensity decreased, the linearity value appeared to increase.

**Figure-6:**
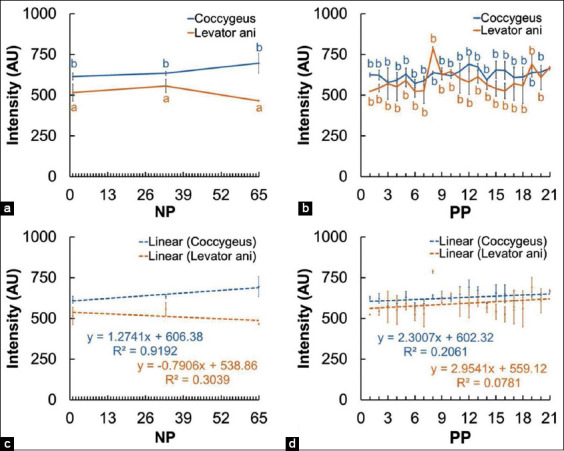
Intensity of the perineal muscles (coccygeus and levator ani) in non-pregnant and postpartum Madura beef cow. a=Perineal muscle intensity of NP cow, b=Perineal muscle intensity of PP cow, c=Linearity of the perineal muscle intensity of NP, d=Linearity of the perineal muscle intensity of PP cow. ^a,b^superscript showed difference (p<0.05).

Figure-6 shows the difference in the mean value of the intensity of the coccygeus and levator ani muscles in both scanning views between NP and PP Madura beef cow. The difference in the intensity of the coccygeus muscle of the PP cow and the NP cow was not significant (p>0.05). However, the difference in the intensity of the levator ani muscle of the PP cow and the NP cow was significant (p<0.05).

**Figure-7:**
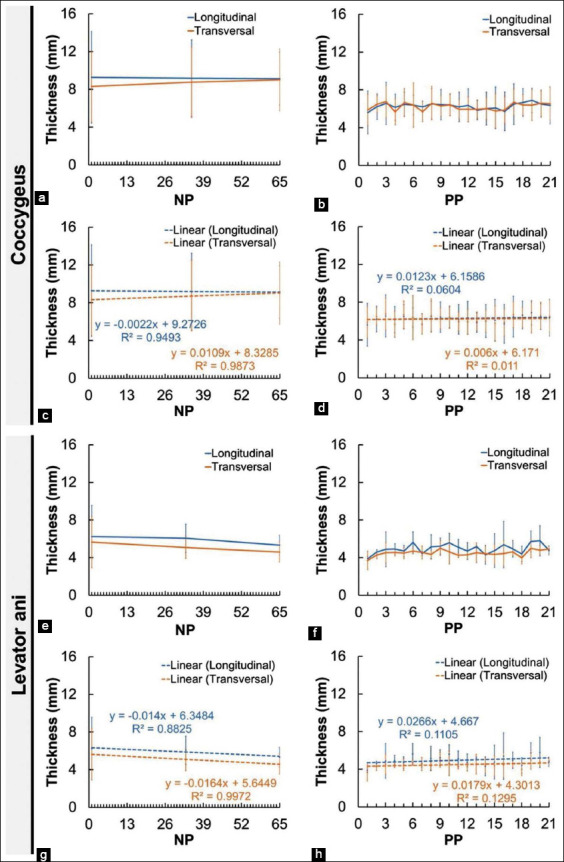
Thickness of the perineal muscles (coccygeus and levator ani muscle) in non-pregnant and postpartum Madura beef cow. a=NP coccygeus muscle thickness, b=PP coccygeus muscle thickness, c=Linearity of NP coccygeus muscle, d=Linearity of PP coccygeus muscle, e=NP levator ani muscle thickness, f=PP levator ani muscle thickness, g=Linearity of NP levator ani muscle, and h=Linearity of PP levator ani muscle.

The thickness of the coccygeus muscle and levator ani muscle of the PP and NP Madura beef cow is shown in Figure-7. NP cow coccygeus and levator ani muscle showed a decrease in diameter. The thickness of the coccygeus muscle in the longitudinal and transverse views at the beginning of observation was 13.3 mm and 13.9 mm, respectively, which decreased to 11.7 mm and 11.5 mm, respectively, at the end of the observation period. The diameter of the levator ani muscle in the longitudinal and transverse views was 11.7 mm and 10.7 mm, respectively, on initial observation, which decreased to 8.8 mm and 9.6 mm, respectively, at the end of the observation period. The thickness of the coccygeus and levator ani muscles of the PP Madura cow increased during the involution process. The coccygeus muscle had a thickness of 7.7 mm and 9.2 mm, respectively, in the longitudinal and transverse views in day 1 PP, which changed to 8.5 mm and 9.0 mm, respectively, on day 21 PP. The thickness of the levator ani muscle in the longitudinal and transverse views was 5.2 mm and 5.7 mm, respectively, in day 1 PP and increased to 7.6 mm and 6.5 mm, respectively, on day 21.

**Figure-8:**
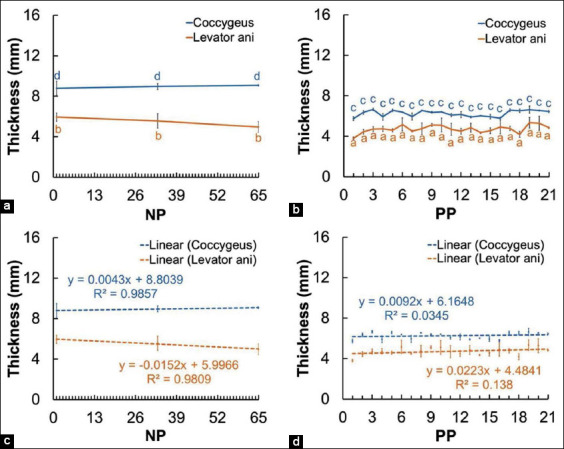
Thickness of the perineal muscles (coccygeus and levator ani) in non-pregnant and postpartum Madura beef cow. a=Thickness of the perineal muscle of NP cow, b=Thickness of the perineal muscle of PP cow, c=Linearity of the perineal muscles of NP cow, d=Linearity of the perineal muscles of PP cow. ^a,b^superscript showed difference (p<0.05).

Figure-8 shows the difference in the mean thickness of the coccygeus and levator ani muscles of the NP and PP Madura beef cow in both scanning views. The difference in thickness of the coccygeus muscle of the PP cow and NP cow is significant (p<0.05). Similarly, there is a significant difference in the thickness of the levator ani muscles of the PP and NP cows (p<0.05).

## Discussion

This research successfully provided imaging of the uterine involution process (Figures-2 and 3) and demonstrated the occurrence of changes in thickness and intensity of the perineal muscles in Madura beef cow using brightness-mode sonogram (Figures-4-8). Caruncle tissue sonogram showed shrinkage and decrease of lochia volume, which was not visible at the end of observation (Figures 2d, p). Cervix, corpus uteri, and cornua uteri shrunk until day 21 (Figures-2b, h, n, d, j, p, f, l, r). Cervix, corpus uteri, and cornua uteri diameter decreased daily during uterine involution (Figure-3c). The gradual decrease in diameter was caused by uterine contraction, tissue shrinkage, caruncle cell death and exfoliation, and endometrium regeneration [15]. Uterine involution that occurred up to day 21 was caused by the return of the uterus to its normal size [17]. However, this change does not appear in histological examination [18] and is instead imaged through USG or other radiological imaging.

The PP period is the period after birth up to the completion of uterine involution [19]. During the PP period, uterine involution occurs along with milk production and resumption of the ovarian cycle influenced by hormones, which requires a relatively large amount of energy. Estrogen is one of the hormones that play an important role in reproductive activities and energy metabolism [20]. Estrogen levels in the PP cow falls until it cannot be detected [21]. Cows that are nursing calf or being milked will undergo a massive consumption of energy reserves. Energy requirements that cannot be supplied through feed will affect the negative energy balance (NEB) [22] and may even cause metabolic diseases, such as hypocalcemia, hyperketonemia, and lipomobilization [23].

The NEB condition will cause an increase in lipolytic and proteolytic activity [24]. Increasing lipolysis and proteolysis can a decreased in lipid and protein composition in muscles and an increase in non-esterified fatty acid (NEFA) concentration to fulfill the demand of nursing cows PP [25]. The ovarium undergoes follicle wave inhibition at the end of the gestation period, and during PP this would restart again. However, ovulation may only occur with the occurrence of the luteinizing hormone peak, which depends on energy balance [19,26]. PP cows may exhibit estrus signs even if it is rarely observed [19]. However, NEB does not give a negative effect in the peak and the length of estrus signs [27] and will influence the weight and BCS [28].

Body weight and BCS, which decrease during NEB period, are indicated by the increase of NEFA concentration [28]. This is strengthened in a research by Wang *et al*. [29], which stated that there is a positive correlation between PP BCS drop and the increase of NEFA concentration. This is also in accordance with a research by Halachmi *et al*. [30] and Singh *et al*. [31] who stated that muscle mass decrease will cause the decrease of BCS. Decreasing body weight and BCS will influence muscle tissue intensity (Figures-5 and 6) and tissue thickness (Figures-7 and 8).

Madura beef cow perineal muscle intensity appeared to decrease during the 21 days of uterine involution (Figures-4d-f, j-l, p-r, v-x). The echogenicity of normal muscle in sonogram will appear hypoechoic [32]. According to Strasser *et al*. [33], a hypoechoic image in the muscle sonogram appeared because the muscle cell is almost anechoic, while fat and fibroblast have higher echogenicity. Intramuscular fat is fat located inside the muscle [34]. The distribution pattern of intramuscular fat differs according to cow breed. Deposition of intramuscular fat by body perineal region is higher in dairy cow compared to meat-producing (beef) cow [35]. Although the perineal muscle of beef cow has lower intramuscular fat, this condition can still be detected by USG (Figure-4).

Muscle intensity depends on the comparison between muscle cell and intramuscular fat [36]. Decreasing intensity of coccygeus and levator ani muscle of PP Madura beef cow was indicated by the decrease in dispersion in the intramuscular fat sonogram (Figures-4d-f, j-l, p-r, v-x). Intramuscular fat muscle sonogram was indicated by infrequent absorbance and dispersion of sound waves based on the dispersion of intramuscular fat and connective tissue [37]. Decreasing muscle echogenicity in the sonogram may be caused by the decrease of fat due to lipolysis and proteolysis [38]. PP cows require a large amount of energy for several body activities, but the estrogen hormone as a key regulator of metabolism is also low. Low estrogen in the body causes a decrease in insulin sensitivity [20], thus triggering the homeostasis of energy metabolism by cells [39]. Homeostatic conditions cause intramuscular fat to be the earliest used in the body’s metabolic processes [40]. The body being in a high-energy requirement condition causes the increased usage of fat as an energy source. Intramuscular fat is the first to be used for body metabolic processes. Intermuscular fat and finally subcutaneous fat will be used after intramuscular fat. However, during the formation of fat deposit, intramuscular fat is the last formed during deposition of excess energy [35]. Early and higher intramuscular fat used in PP cow and most recently in the deposition process can be the cause of significant differences intensity value (p<0.05) between the levator ani muscle of Madura PP cow and Madura NP cow (Figures-6a and b).

Muscle intensity depends on the comparison between muscle cell and intramuscular fat. Perineal muscles will undergo a decrease in intensity caused by lower intramuscular fat as opposed to muscle cells (Figures-4d-f, j-l, p-r, v-x). The formation of intramuscular fat depends on genetics, environment, and nutrition [41]. The balance of nutrition supply in cow in the prepartum and PP periods influences the availability of intramuscular fat. It has been reported that nutrition fulfillment through the foraging feeding system for 4 months does not show an increase in intramuscular fat deposition. Intramuscular fat deposition is increased following a high protein source ration [35]. The availability of intramuscular fat is required during the PP period, which will influence the reproductive cycle of the next period. According to a research by Boyles [42], a higher sonogram score of the longissimus muscle intramuscular fat in *Bos Taurus* cow has a positive correlation with gestation percentage. The sonogram of the perineal muscles of PP Madura beef cow (Figure-4), especially the intensity of the levator ani muscles (Figures-6b and d), shows the need for efforts to maintain intramuscular fat so that it does not decrease during PP by supplementing nutrition.

The difference in muscle intensity of the coccygeus and levator ani in different scanning views, namely, the longitudinal and transverse views (Figures-4 and 5), can occur due to the morphometric differences in intramuscular fat content analysis [36]. The intensity of the coccygeus and levator ani muscles of Madura beef cows showed significant differences in the NP group (p<0.05); however, this was not significant in the PP group (p>0.05) (Figure-6). The difference in the intensity of the coccygeus and levator ani muscles can also be caused by the physicochemical differences between these muscles [43].

The decreasing intensity of the coccygeus and levator ani muscles of the Madura beef cow was followed by an increase in muscle thickness (Figures-5b, 5f, 6b, 6d, 7b, 7f, 8b, and 8d). However, this is different with the decreasing thickness of the coccygeus and levator ani muscles of NP Madura beef cow (Figures 7a and e). The decreasing thickness of the coccygeus and levator ani muscles in NP Madura beef cow occurred due to muscle volume decrease caused by the increase of metabolism in the muscle during normal condition, neither gestating nor in parturition (Figures-7a and e). Changes in muscle thickness can also be caused by changes in the diameter and length of the muscle fibers. Furthermore, changes in the muscle fibers are more influenced by the availability of nutrients in the body to meet energy needs [44].

The increasing thickness of the coccygeus and levator ani muscles in PP Madura beef cow is caused by the muscles returning to normal size (Figures-7b and f). The thickness of the coccygeus muscle in PP Madura beef cow increased by 0.78 mm and decreased by 0.13 mm in the longitudinal and transverse views, respectively (Figure-7b). Although at the end of observation, the coccygeus muscle thickness in the longitudinal view appeared to be lower, in linearity, a slope value above 0 was obtained (Figure-7d). This indicates that the coccygeus muscle tends to be increased in the transverse view. The increase in coccygeus muscle thickness in Madura beef cow is similar to that of PP dairy cow [13]. The levator ani muscle also underwent an increase in thickness of 2.46 mm and 0.72 mm in the longitudinal and transverse views, respectively (Figure-7f). The sonogram of the perineal muscles (coccygeus and levator ani) showed a decrease in size (Figures-7b, f, and 8b) in the early PP period still influenced by muscle relaxation caused by the high concentration of relaxin hormone during the prepartum and partum period [45]. The increase in the concentration of estrogen few days PP can lead to an increase in muscle thickness [46].

The increasing thickness of perineal muscles in PP Madura beef cow is not only caused by a decrease of relaxin hormone [9,10] but also by the change of weight burden with the decrease in uterus weight during the PP period. The decrease in uterus weight is indicated by the size of the reproductive tracts, such as cervix, corpus uterus, and cornua uterus, which returned to the initial size during PP (Figure-2). Cervix and corpus size on day 21 decreased as much as 35.6 mm and 44.67 mm, respectively, and cornua uterus size decreased as much as 16.93 mm for 11 days (day 10 up to day 21) (Figure-3c). This condition causes the lowering traction of the perineal diarrhea muscle, which allows the muscle to return to normal size after the relaxation during parturition. The thickness of the coccygeus muscle of the PP cow increased by 0.70 mm and the levator ani increased by 1.03 mm (Figure-8b). The difference in the thickness of the coccygeus and levator ani muscles can also be caused by differences in the diameter and length of the muscle fibers (Figure-8b). The increase in the coccygeus and levator ani muscle thickness in Madura cow is similar to that of PP dairy cow [13].

Anal triangle muscles composed of the coccygeus, levator ani, and external anal sphincter compose an interconnecting muscle tissue system. The urogenital tract and anus are connected by the perineal body (centrum tendineum perinei) [47]. The levator ani muscle of PP Madura beef cow appeared to have a higher size increase compared to coccygeus muscle during uterine involution (Figures-7b and f). The levator ani muscle is directly connected to the external anal sphincter muscle, which connects to the rectum [48]. Its closer proximity to the external anal sphincter compared to the coccygeus muscle caused it to receive a different weight burden during muscle contraction throughout gestation and muscle relaxation in parturition [13]. This difference in weight burden from the reproductive tract caused the difference in thickness between the coccygeus and levator ani muscles in Madura beef cow, causing them to shrink during involution. This could be the cause of a significant difference in the thickness of the coccygeus and levator ani muscles between NP and PP Madura beef cow.

The coccygeus muscle and levator ani muscle of the Madura beef cow in both the NP and PP group showed different intensity and thickness in the longitudinal and transverse view (Figures-5a, b, e, f and 7a, b, e, f). The difference in intensity of the coccygeus and levator ani muscles could have been caused by physicochemical differences between the two [43]. The difference in muscle intensity between the different views may be caused by their difference in the morphometric intramuscular fat concentration [36]. The difference in thickness between longitudinal and transverse views could be caused by probe positioning during imaging. Based on a research by Santos and Armada-da-Silva [49], the average quadriceps femoris muscle thickness in the longitudinal view differs with of the transverse view. This difference could also be seen from the medialis and lateralis region of both views. However, McCreesh and Egan [50] differ in how they stated that there is no significant difference in thickness between longitudinal and transverse views.

During the delivery process, the fetus passes through the uterus, cervix, vagina, and vulva, which are in the pelvic space [51]. Therefore, the contraction and dilation in the labor process greatly affect the muscles in the reproductive organs and the pelvic space. This can cause trauma to the muscle tissue in the organs and attached to the pelvic bones. However, in parturition cows, trauma often occurs in the vaginal and vulvar muscles, such as in vaginal prolapse [52], and no research results have been available on trauma in the coccygeus and levator ani muscles in cows.

## Conclusion

The involution process in cows is indicated by the decrease in reproductive organ size, which influences perineal muscles in the form of decreasing intensity and increasing thickness, both of which can be well-observed using USG imaging.

## Availability of Data and Materials

All data generated or analyzed during this study are included in this published article.

## Authors’ Contributions

MFU and SYH conceptualized, designed, analyzed the results, and researched the literature. SYH conducted the research, collected, and processed the data. MFU, AA1 and AA2 supervised the research and critical review. SYH, MFU, AA1 and AA2 wrote the manuscript.
